# Comparison of the Aptima HIV‐1 Quant Dx assay with the COBAS AmpliPrep/COBAS TaqMan HIV‐1 v2.0 Test for HIV‐1 viral load quantification in plasma samples from HIV‐1–infected patients

**DOI:** 10.1002/hsr2.31

**Published:** 2018-03-13

**Authors:** Serena Longo, Isabella Bon, Giuseppina Musumeci, Alessia Bertoldi, Vanessa D'Urbano, Leonardo Calza, Maria Carla Re

**Affiliations:** ^1^ Microbiology Section of the Department of Experimental, Diagnostic and Specialty Medicine, School of Medicine University of Bologna Italy; ^2^ Clinics of Infectious Diseases, Department of Medical and Surgical Sciences St. Orsola‐Malpighi Hospital, University of Bologna Italy

**Keywords:** HIV‐1, HIV‐1 RNA quantitation, HIV‐1 subtypes, clinical samples

## Abstract

**Background and aims:**

HIV‐1 RNA viral load (VL) in plasma samples of HIV‐1–positive patients is used to assess the level of viral replication, the risk of disease progression, and the response and efficacy to antiretroviral treatment. Knowing the performance of different tests for HIV‐1 RNA detection is, therefore, important for clinical care. This study compared the performance of the recently introduced Aptima HIV‐1 Quant Dx assay (Hologic, Inc) and the standard COBAS AmpliPrep/COBAS TaqMan HIV‐1 v2.0 Test (CAP/CTM2) (Roche Molecular System, Inc) for HIV‐1 RNA quantitation.

**Methods:**

Assay performance was assessed using 335 clinical samples, a standard HIV‐1 low VL panel, and 2 diluted samples from well‐characterized patients infected with different HIV‐1 subtypes tested in 5 replicates over 3 days. All samples were tested on both assays to evaluate inter‐assay agreement, both qualitatively and quantitively. Altogether, we evaluated assay sensitivity, linearity, accuracy, precision, repeatability, and reproducibility.

**Results:**

Assay agreement for qualitative results in 335 clinical samples was fair (80.6%). Correlation of quantitative assay results (n = 164) was excellent (R ^2^ = 0.97), with 96.3% of the results within the 95% limit of assay agreement (−0.42 to +0.86 log), and 98.8% within 1 log of each other. Aptima‐HIV‐1 yielded results, on average, 0.22 log higher than CAP/CTM2. Both assays accurately quantitated the HIV‐1 standard at low VL (R ^2^ ≥ 0.94), with all samples within 0.5 log of the target.

**Conclusion:**

Aptima‐HIV‐1 assay demonstrated sensitivity, accuracy, reproducibility, and precision for the detection and quantitation of HIV‐1 RNA across a wide dynamic range of VLs. Its performance, together with full automation and high throughput, suggests that Aptima‐HIV‐1 could be a suitable assay for reliable monitoring of HIV‐1 VL in patients undergoing treatment.

## Abstract 2

**Background and aims**

HIV‐1 RNA viral load (VL) in plasma samples of HIV‐1–positive patients is used to assess the level of viral replication, the risk of disease progression, and the response and efficacy to antiretroviral treatment. Knowing the performance of different tests for HIV‐1 RNA detection is, therefore, important for clinical care. This study compared the performance of the recently introduced Aptima HIV‐1 Quant Dx assay (Hologic, Inc) and the standard COBAS AmpliPrep/COBAS TaqMan HIV‐1 v2.0 Test (CAP/CTM2) (Roche Molecular System, Inc) for HIV‐1 RNA quantitation.

**Methods**

Assay performance was assessed using 335 clinical samples, a standard HIV‐1 low VL panel, and 2 diluted samples from well‐characterized patients infected with different HIV‐1 subtypes tested in 5 replicates over 3 days. All samples were tested on both assays to evaluate inter‐assay agreement, both qualitatively and quantitively. Altogether, we evaluated assay sensitivity, linearity, accuracy, precision, repeatability, and reproducibility.

**Results**

Assay agreement for qualitative results in 335 clinical samples was fair (80.6%). Correlation of quantitative assay results (n = 164) was excellent (R ^2^ = 0.97), with 96.3% of the results within the 95% limit of assay agreement (−0.42 to +0.86 log), and 98.8% within 1 log of each other. Aptima‐HIV‐1 yielded results, on average, 0.22 log higher than CAP/CTM2. Both assays accurately quantitated the HIV‐1 standard at low VL (R ^2^ ≥ 0.94), with all samples within 0.5 log of the target.

**Conclusion**

Aptima‐HIV‐1 assay demonstrated sensitivity, accuracy, reproducibility, and precision for the detection and quantitation of HIV‐1 RNA across a wide dynamic range of VLs. Its performance, together with full automation and high throughput, suggests that Aptima‐HIV‐1 could be a suitable assay for reliable monitoring of HIV‐1 VL in patients undergoing treatment.

## 1 INTRODUCTION

The introduction of new antiretroviral agents in the last decade has significantly improved the efficacy and safety of antiretroviral therapy (ART) in HIV‐1–infected patients.^1^ Besides clinical and immunological monitoring, which are used as complementary evaluations, HIV‐1 RNA quantitation in patients' plasma samples is currently considered the main approach to monitor ART compliance and success.^2^, ^3^, ^4^

Optimal control of HIV‐1 infection is reached when the complete viral suppression achieved persists over time. Even if full viral suppression is achievable in most patients (both treatment‐naïve and experienced), some show a transient, low viremia (“blips”).^5^, ^6^ Some blips might be considered artifactual variations in viral load (VL) because of assay variability and laboratory processing inconsistencies,^7^ not associated with an increased risk of treatment failure or drug resistance. Key treatment decisions made at VLs ranging from 1.7 to 3 log copies/mL need accurate monitoring in clinical samples and, consequently, require a highly sensitive, precise, and reproducible HIV‐1 RNA quantitation assay. Other important attributes of a VL assay include the ability to quantitate HIV‐1 RNA precisely over a wide range of VLs and an equally good performance on all HIV‐1 subtypes. As assay agreement on quantitative values is generally good at high VLs but tends to decrease substantially at low VLs,^8^, ^9^, ^10^, ^11^ the performance characteristics of an assay able to detect low VLs should be taken into account when making clinical decisions.

This study compared the performance characteristics of the recently introduced CE‐(2015) Aptima HIV‐1 Quant Dx assay^12^ and the routinely used Roche COBAS AmpliPrep/COBAS TaqMan HIV‐1 v2.0 Test (CAP/CTM2).^13^

## 2 MATERIALS AND METHODS

### 2.1 Clinical samples

The study included clinical samples obtained from HIV‐1–infected patients attending the Infectious Diseases Unit, St. Orsola Hospital, Bologna, Italy, for routine monitoring of HIV‐1 VL (January 2014‐May 2017). The study was approved by the local ethics committee (737/2016), and the patients provided written informed consent.

#### 2.1.1 HIV‐1 samples

The 335 plasma samples collected in EDTA tubes were tested side‐by‐side in the Aptima‐HIV‐1 and CAP/CTM2 assays, without further criteria for selection other than available sample volume.

Tubes were centrifuged for 10 minutes at 1000 to 3000 *g* for plasma preparation. All samples were first tested with the CAP/CTM2 assay.

If the residual plasma volume was ≥1.2 mL, the same samples were immediately tested in primary tubes on the Hologic Panther instrument. For samples with less than 1.2 mL residual plasma volume, 0.70 mL plasma was transferred to Hologic specimen aliquot tubes.

Among the samples evaluated in the study, 248 specimens were derived from HIV‐1 patients infected with B HIV‐1 strains and 87 samples from other subtypes (A, C, F, G, and CRFs), characterized by phylogenetic analysis of HIV‐1 *pol* gene (RT and PR).^14^, ^15^ In particular, 12 samples belonged to subtypes A, 9 to subtypes C, 23 to subtypes F, 14 to subtypes G, and 29 were circular recombinant forms (CRFs).

### 2.2 HIV‐1 VL assays

Samples in the 2‐assay platforms were processed and tested by trained operators, by Aptima HIV‐1 Quant Dx assay (cat. no. PRD‐03000) and Roche CAP/CTM2 (cat. no. 05212294190) according to the assay manufacturers' package inserts.

### 2.3 Aptima‐HIV‐1 assay

All the samples were tested in specimen aliquot tubes. Samples were loaded onto the Panther system (Hologic, Inc). HIV‐1 genomic RNA was first released using target capture technology and then bound to magnetic particles. The Aptima HIV‐1 Quant assay uses the TMA method to amplify 2 regions of HIV‐1 RNA (*pol* and LTR) from the sample and amplifies and detects the amplified targets, all in an automated manner.

The assay's reported lower limit of quantification (LLOQ) is 1.47 log copies/mL, and its upper limit of quantitation is 7 log copies/mL (Hologic Inc, PI). The reported limit of detection (LoD) of the Aptima‐HIV‐1 assay is 12 cp/mL. Panther system allows random access testing of various analytes, processing up to 275 samples in an 8‐hour shift. The system provides results from 120 samples in about 2.5 hours.

### 2.4 CAP/CTM2 assay

All the samples were tested in Roche S‐tubes. The sample volume used was 1 mL. Tubes were loaded onto the Cobas Ampliprep instrument, which extracts HIV‐1 LTR and *gag* targets from the sample. Tubes were then transferred to the COBAS Taqman Analyzer (Roche Molecular Systems, Inc, cat. no. 03121453001), which amplifies and detects the target sequence in an automated fashion. The reported assay's LLOQ is 1.39 log copies/mL, and its upper limit of quantitation is 7 log copies/mL (Roche Inc. PI). The reported LoD of this assay is 20 cp/mL. The CAP/CTM platform has an initial capacity for 72 samples with continuous feeding, which allows 168 samples (1 mL/sample) to be processed per 8‐hour shift. This system returns results in 4.5 hours.

### 2.5 Assay assessment using an external quality panel by Aptima‐HIV‐1 assay

The Acrometrix HIV‐1 linearity panel (ThermoFisher Scientific, Benicia, California, cat. no. 950470) was used to evaluate both the assays' linearity and accuracy of results at low VLs. The 5 panel members at nominal concentrations of 0, 1.22, 1.52, 1.82, 2.22, and 2.52 log copies/mL were tested in replicates of 5 in each assay.

### 2.6 Assay evaluation in 2 clinical samples (subtypes B and F) by CAP/CTM2 assay and Aptima‐HIV‐1 assay

2 samples with different subtypes (subtype B, subtype F) obtained from HIV‐1 patients previously well characterized^14^, ^15^ were serially diluted to 4 target concentrations (about 2.5 to about 5.5 log copies/mL). Five replicates of each dilution were tested in the Aptima‐HIV‐1 assay on 3 separate days, after storage at 4°C.

### 2.7 Data analyses

VL values were expressed as log copies/mL. Agreement of the assays' qualitative results (ie, defining samples as “negative,” “detected <LLOQ,” and “quantitated”) was determined using a tabular format. For a very small number of plasma samples with discordant results between the 2 assays (eg, negative or detected <LLOQ in one assay and quantitated in the other assay), the patients' immunological data CD4 count and CD4/CD8 ratio, determined as previously described,^15^ were considered.

Clinical samples yielding quantitative values in both assays served to determine the correlation between the paired assay quantitative values. All statistical analyses were performed using GraphPad Prism v.6 (GraphPad Software, Inc, San Diego, California). The correlation was determined by Deming regression analysis with generation of Pearson's correlation coefficient (*R* ^2^), as well as Bland‐Altman analysis and calculation of the average difference between assay results (ie, bias), the limit of agreement between assay results, and the proportion of samples with paired results within 1 log copies/mL of each other.

Accuracy was evaluated by comparing assay results with target values. Linearity of the assay was evaluated by linear regression analysis of the assay results versus the target concentrations. Assay precision was evaluated by calculating the standard deviation (SD) and coefficient of variation (%CV) over the replicates tested.

## 3 RESULTS

### 3.1 Assay performance comparison in clinical samples

Assay performance was compared using all 335 clinical samples (248 HIV‐1 type B and 87 HIV‐1 non‐B samples) with VLs identified by CAP/CTM2, ranging from undetectable (105/335) to detectable HIV‐1 RNA amounts (up to 7 log copies/mL).

As shown in Table 1, assay agreement for qualitative results (undetected, detected <LLOQ, and quantitated) was obtained in 270 out 335 samples (80.6%). Overall, 164 samples showed detectable results in both assays. VL was detected <LLOQ by both assays in 32 samples.

**Table 1:** Concordance of assay results in classifying samples as negative, detected, or quantitated, in clinical samples

CAP/CTM2	Aptima‐HIV			Total
	Not Detected	Detected <30 copies/mL	Quantitated	
Not detected	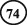	31	0	105
Detected <20 copies/mL	14	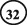	0	46
Quantitated	2	18	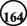	184
Total	90	81	164	335

The numbers in circles indicate the agreement between the assays' qualitative results.

**Figure 1:**
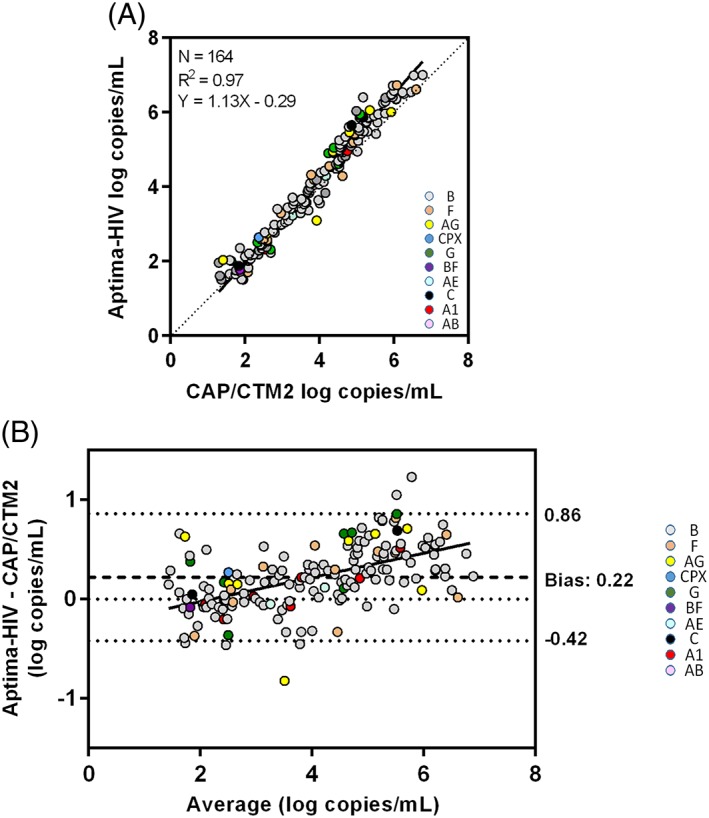
Correlation of assay results in patients' clinical samples quantified by both assays. A, Deming regression analysis and correlation; the dotted line represents identity of assay results. B, Bland‐Altman analysis of agreement between assay results. Dashed line represents the bias; dotted line represents the assays' limit of agreement. HIV subtypes are presented as colored circles

Quantitative results (n = 164) were obtained in both assays with VLs ranging from 1.4 to 7.0 log copies/mL. Quantitative assay values were highly correlated (*R* ^2^: 0.97) (Figure 1A). Assay performance was checked in plasma samples, with VL detected by both assays, from HIV‐1 patients infected with either HIV‐1 B strains (114 samples) or not B HIV‐1 strains (50 samples). Results showed an optimal correlation, regardless of the HIV‐1‐subtype, as demonstrated by Deming regression analysis (Figure 1A**)**.

Only 2 samples **(**Figure 1B), with high VL (>5 log copies/mL), showed VL values differing by >1 log copies/mL between the assays. On average, Aptima‐HIV‐1 tended to produce VL values 0.22 log higher than CAP/CTM2 values. However, this trend was not reflected consistently across the range of VLs, as Aptima‐HIV‐1 values were lower than CAP/CTM2 values for samples with VLs <2 log copies/mL (by 0.05 log) and greater than CAP/CTM2 values for VLs ≥2 log (Figure 1B). Absolute differences between assay quantitative values were the highest (0.49 log copies/mL) for samples with high VLs (>5 log copies/mL) and lowest (0.02 to 0.05) for samples with VLs <4 log copies/mL (data not shown). Overall, 96.3% (158/164) of the results were within the 95% limit of agreement of the assays (−0.42 to +0.86 log copies/mL) (Figure 1B). Of the 6 outlier samples (outside the limit of agreement), three differed by <0.5 log in both assays, one differed by >0.5 log but <1 log, and two by >1 log but <1.5 log.

As shown in Table 1, 65 samples showed different results, more pronounced when HIV‐1 VL was relatively low. In particular, 31 samples, undetected by CAP/CTM2, showed a low HIV‐1 RNA amount (<LLOQ) by Aptima‐HIV‐1, and 14 samples, undetected by Aptima‐HIV‐1, showed low VL amount (<LLOQ) by CAP/CTM2. In addition, among the remaining 20 samples with discordant results, 2 samples quantitated by CAP/CTM2 showed undetectable VL by Aptima‐HIV‐1, and 18 samples, quantitated by CAP/CTM2, showed HIV‐1 RNA levels <LLOQ by Aptima‐HIV‐1.

Among these 20 samples with discordant results (Table 2A), 14 showed <30 copies/mL HIV‐1 RNA by Aptima and detectable viral replication under 50 copies/mL (ranging from 31 to 47 copies/mL) by CAP/CTM2. The remaining 6 samples (patients 15‐20) presented <LLOQ levels (sample 15‐18) or undetectable results by Aptima‐HIV‐1 (patients 19‐20), and VLs ranging from 53 to 255 by CAP/CTM2.

**Table 2A:** Analysis of 20 discordant samples quantitated by CAP/CTM2, but either “not detected” or “detected <30 copies/mL” by Aptima‐HIV

	HIV‐1 Subtype	Aptima‐HIV‐1	CAP‐CTM2
Patient 1	*C*	<30	31
Patient 2	*B*	<30	31
Patient 3	*A*	<30	32
Patient 4	*B*	<30	32
Patient 5	*B*	<30	35
Patient 6	*B*	<30	38
Patient 7	*F*	<30	40
Patient 8	*B*	<30	42
Patient 9	*F*	<30	44
Patient 10	*B*	<30	45
Patient 11	*A*	<30	47
Patient 12	*B*	<30	42
Patient 13	*G*	<30	41
Patient 14	*B*	<30	47
Patient 15	*B*	<30	84
Patient 16	*B*	<30	102
Patient 17	*B*	<30	103
Patient 18	*C*	<30	113
Patient 19	*B*	TND	255
Patient 20	*C*	TND	53

Highlighted in gray are the CAP/CTM2 results, which are discordant with the Aptima‐HIV result.

Abbreviation: TND, target not detected.

Particular attention was given to these last 6 samples (Table 2A, B), for which corresponding immunological data (CD4, CD8, and CD4/CD8 ratio) was obtained during follow‐up. For patients 17, 18, and 19, the CAP/CTM2 results might be interpreted as a viral blip, even if not justified by the CD4 cell count that seem to be stable over time. Moreover, blood samples from patients 15, 16, and 20 exhibited a detectable value of VL (84, 102, and 113 copies/mL, respectively), as determined by CAP/CTM2 VL results, accompanied, in further samples, by a clear improvement in immunological parameters.

**Table 2B:** For patients 15‐20, the discrepancy was further analyzed by providing patients' longitudinal clinical and VL data

Patient No.	Dates of VL Measurements (Month, Day, Year)						
Patient 15	12.05.2014	09.09.2015	02.02.2016	11.05.2016	09.02.2016		
CD4 (cells/μL)	772	531	502	630	876		
CD4/CD8 ratio	0.97	0.79	0.81	0.97	1.35		
CAP/CTM2VL(cp/mL)	6723	25470	23479	84	TND		
Patient 16	09.14.2015	07.12.2015	03.30.2016	08.03.2016	01.16.2017		
CD4 (cells/μL)	90	131	201	257	305		
CD4/CD8 ratio	0.26	0.29	0.23	0.36	0.27		
CAP/CTM2VL(cp/mL)	55	64	102	23	<20		
Patient 17	10.30.2015	02.16.2016	06.14.2016	08.25.2016	11.02.2016	02.08.2017	
CD4 (cells/μL)	10	158	196	193	227	231	
CD4/CD8 ratio	0.14	0.18	0.12	0.16	0.24	0.16	
CAP/CTM2VL(cp/mL)	<20	<20	103	<20	<20	<20	
Patient 18	02.20.2015	06.23.2016	10.07.2015	12.19.2016	06.17.2016	08.25.2016	12.13.2016
CD4 (cells/μL)	166	239	258	256	308	263	280
CD4/CD8 ratio	0.25	0.26	0.30	0.22	0.22	0.26	0.30
CAP/CTM2VL(cp/mL)	<20	128	168	94	113	<20	TND
Patient 19	10.01.2015	12.09.2015	01.21.2016	05.02.2016	06.22.2016	09.07.2016	
CD4 (cells/μL)	20	24	170	176	175	195	
CD4/CD8 ratio	0.48	0.53	0.57	0.60	0.65	0.75	
CAP/CTM2VL(cp/mL)	TND	TND	TND	255	TND	TND	
Patient 20	12.14.2015	02.24.2016	04.06.2016	06.15.2016	07.20.2016	08.30.2016	
CD4 (cells/μL)	1840	1925	2072	1344	1810	1661	
CD4/CD8 ratio	1.6	2.0	1.65	1.66	1.66	1.82	
CAP/CTM2VL(cp/mL)	TND	TND	TND	53	TND	TND	

Highlighted in gray are the CAP/CTM2 results, which are discordant with the Aptima‐HIV result.

Abbreviations: cp/mL, copies/mL; TND, target not detected.

#### 3.1.1 HIV‐1 subtypes

Results obtained by the analysis of 87 samples from HIV‐1 patients infected with subtypes A, C, F, G, and CRFs showed that similar findings could be obtained from samples derived from HIV‐1–infected subjects with subtypes A and C, irrespective of HIV‐1 RNA amounts, with no significant difference between the assays used. On the other hand, Aptima HIV‐1 Quant DX assay was able to detect higher level of viral replication in samples containing HIV‐1 subtype F, G, and CRFs, revealing important differences (≥0.5 log) in 13 samples (5 belonging to subtype F, 3 to subtype G, 5 to CRFs), as shown in Table 3.

**Table 3:** Different levels of viral replication obtained by Aptima HIV‐1 Quant DX and CAP/CTM2 in 13 samples from HIV‐1 patients infected with different HIV subtypes (F, G, and CRFs)

Sample No.	HIV‐1 Subtype	CAP/CTM2		Aptima‐HIV‐1	
		cp/mL	log cp/mL	cp/mL	log cp/mL
Sample A	*G*	121.866	5,09	897.480	5,95
Sample B	*F*	124.296	5,09	814.929	5,91
Sample C	*CRF12_BF*	72.139	4,86	460.368	5,66
Sample D	*CRF_AG*	226.153	5,35	1.138.495	6,06
Sample E	*F*	151.781	5,18	746.774	5,87
Sample F	*G*	24.052	4,38	111.580	5,05
Sample G	*CRF_AG*	63.500	4,80	291.068	5,46
Sample H	*F*	1.189.204	6,08	5.423.568	6,73
Sample I	*G*	17.537	4,24	78.952	4,90
Sample J	*CRF01_AG*	26	1,41	109	2,04
Sample K	*CRF01_AG*	22.727	4,36	88.747	4,95
Sample L	*F*	6.057	3,78	21.128	4,32
Sample M	*F*	206.830	5,32	697.513	5,84

Abbreviation: cp/mL, copies/mL.

### 3.2 Assay accuracy with a standard panel

**Figure 2:**
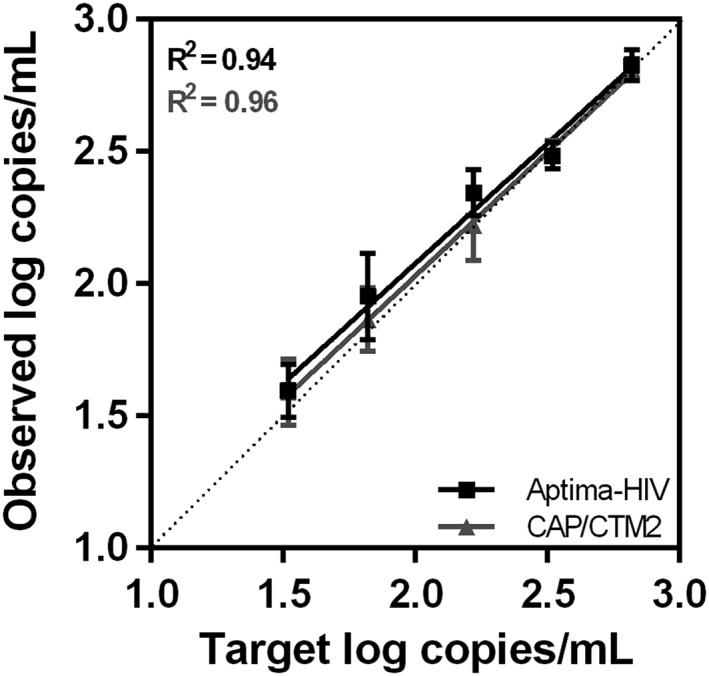
Assay results with the Acrometrix HIV‐1 panel. The mean values of 5 replicates are plotted with the error bars representing the SD. The dotted line represents identity of assay values with target values

The assays' ability to accurately quantitate HIV‐1 RNA at low VLs was evaluated using the Acrometrix standard at target concentrations ranging from 1.2 to 2.8 log copies/mL. Assay results showed a very good precision, with all assay results differing by <0.5 log copies/mL from the target values, and excellent linearity (*R* ^2^ ≥ 0.94) (Figure 2).

### 3.3 Repeatability, reproducibility, precision, and linearity with patient's samples of 2 subtypes

Two well‐characterized^14^, ^15^ clinical samples (subtype B and F) were tested by Aptima‐HIV‐1 to determine within‐run repeatability, between‐run reproducibility, precision, and linearity of the assay. Within‐run repeatability was substantially good, as reflected by an SD ≤0.16 for the 5 replicates tested (Table 4). Between‐run repeatability was good, with 23 of the 24 results, obtained on 3 different days, being within 0.5 log of each other (results for the subtype F at low VL on day 1 differed by >0.5 but <1 log from results on day 2 or 3). Overall precision of the assay was excellent, with 23/24 %CV <4% and all %CVs being <8%.

**Table 4:** Aptima‐HIV results for 2 clinical samples (4 dilutions) tested in 5 replicates in 3 different days

	HIV‐1 Subtype B				HIV‐1 Subtype F			
	Target^a^	Average Aptima^b^	SD	%CV	Target^a^	Average Aptima^b^	SD	%CV
Day 1	5.50	5.46	0.12	2.25	5.62	5.63	0.09	1.64
	4.50	4.35	0.16	3.74	4.62	4.08	0.14	3.33
	3.50	3.29	0.06	1.71	3.62	3.11	0.08	2.42
	2.50	2.53	0.10	3.88	2.62	1.85	0.15	7.89
Day 2	5.50	5.38	0.03	0.49	5.62	5.61	0.02	0.40
	4.50	4.28	0.02	0.46	4.62	4.51	0.10	2.12
	3.50	3.35	0.03	0.81	3.62	3.50	0.07	1.97
	2.50	2.53	0.08	3.13	2.62	2.62	0.06	2.27
Day 3	5.50	5.06	0.03	0.55	5.62	5.50	0.04	0.77
	4.50	3.97	0.15	3.67	4.62	4.44	0.13	3.02
	3.50	3.12	0.16	5.23	3.62	3.37	0.11	3.15
	2.50	2.34	0.06	2.61	2.62	2.54	0.07	2.68

All data except %CV are in log copies/mL.

a Target value determined by historical CAP/CTM2 results.

b Average of 5 replicates.

Accuracy was also excellent, with 22 of 24 results being within 0.5 log of the target, and 2 results differing by >0.5 but <1 log from the target. Linearity was excellent, as demonstrated by all correlation coefficients >0.98 (data not shown).

## 4 DISCUSSION

As HIV‐1 VL monitoring has become the cornerstone for the management of HIV‐1‐positive patients during their lifelong treatment regimen(s), the assays used to measure HIV‐1 VL must be highly sensitive, specific, accurate, and precise. In the present study, the TMA‐based Aptima‐HIV‐1 assay demonstrated sensitivity, reproducibility, and precision for the detection and quantitation of HIV‐1‐RNA across a wide dynamic range of VLs (including very low VLs).

Assay agreement for qualitative results in 335 clinical samples was fair (80.6%). Quantitative results for Aptima‐HIV‐1 and CAP/CTM were highly correlated (*R* ^2^ = 0.97) and only 2 of 164 samples quantitated by both assays had results that differed by >1 log copies/mL but <1.5 log copies/mL. Although Aptima‐HIV‐1 results were on average slightly higher than CAP/CTM2 results (by 0.22 log), the difference between assay results was minimal at low VLs (0.05 at VLs 1‐1.99 log; 0.04 at VLs 2‐2.99 log, and 0.02 at VLs 3‐3.99 log).

Among the 65 discordant results, most samples did not show substantial variations. Indeed, in 45 samples the differences were very small (less than 30 or 20 copies/mL by one test and undetectable by the other test) and may not be considered as *real conflicting* results. In fact, optimal viral suppression is generally defined as a VL persistently below the level of detection (HIV RNA <20 to 75 copies/mL, depending on the assay used).^2^

On the other hand, 20 samples quantitated by CAP/CTM2, but either undetected or detected <LLOQ in Aptima‐HIV‐1, deserved further analysis. For 14 of these samples, CAP/CTM2 values were <50 copies/mL, while Aptima‐HIV‐1 results were <30 copies/mL, a difference that could considered not to be clinically significant since consensus threshold defines viral suppression as <50 copies/mL.^2^

Among the 6 blood samples only detectable by CAP‐CTM2 (VL >50 HIV‐1 RNA copies/mL, ranging from 53 to 255 copies/mL), results obtained during follow‐up showed VL values reported as *Target not detectable* or very low (<30 copies/mL). In all of these cases, CD4 values were stable, suggesting that results had to be globally evaluated considering both VL and CD4 values. However, when a detectable VL of 50 to 400 copies/mL is preceded or followed by an undetectable result, testing the sample again is recommended to avoid an assay artifact or to establish a true viral rebound.^16^, ^17^, ^18^

Moreover, the occurrence of so called viral blips (50‐400/1000 copies/mL) during treatment are important events, which could be misinterpreted as treatment failure and hence may lead to a change in medication, since the goal of ART is VL suppression to TND or <50 copies/mL.

While we cannot rule out the probability that proviral HIV‐1 genomic sequences in the plasma could be responsible for the blips (eg, originating from latently infected cells in the pellet), the small number of samples prevents any definitive conclusion.

Our results concur with those of other studies that found the Aptima‐HIV‐1 assay to have a performance comparable with the CAP/CTM2 test.^19^, ^20^, ^21^, ^22^, ^23^, ^24^, ^25^, ^26^ Several data^19^, ^20^, ^21^, ^22^, ^25^ showed a small (<0.23 log) positive bias for Aptima‐HIV‐1 VLs, whereas one study found a small (0.075) negative bias.^23^ Yet another study found Aptima‐HIV‐1 to be more sensitive than CAP/CTM2.^27^ Aptima‐HIV‐1 has also been shown to have performance characteristics similar to^19^, ^28^, ^29^ or better^30^ than the RealTime HIV‐1 assay (Abbott Molecular, Des Plaines, Illinois) and superior to the NucliSens EasyQ HIV‐1 v.2 assay (BioMérieux, Marcy l'Etoile, France)^28^ and the Artus HIV‐1 QS‐RGQ assay (Qiagen GmbH, Hilden, Germany).^19^

Finally, Aptima's performance was equally good in B and non‐B subtypes, including CRFs, as documented by other studies.^20^, ^21^, ^24^, ^28^, ^29^

The Aptima assay demonstrated good performance, sensitivity, precision, and reliability, in addition to an excellent clinical agreement. Combined with full automation, high throughput, and superior workflow,^31^ Aptima‐HIV‐1 is suitable for VL monitoring of HIV‐1 patients during treatment.

## FUNDING

This work was supported by Finalized and oriented research, University of Bologna Italy (Funds 2015 and 2016) and Fanti Melloni Foundation, Bologna, Italy (Funds 2015).

## CONFLICT OF INTERESTS

The authors declare that they have no conflict of interests.

## ETHICAL APPROVAL

The study was approved by the local ethics committee (737/2016), and the patients provided written informed consent.

## AUTHOR CONTRIBUTIONS

Conceptualization: Serena Longo, Maria Carla Re

Data curation Isabella Bon, Giuseppina Musumeci

Investigation: Leonardo Calza

Formal analysis: Alessia Bertoldi, Vanessa D’ Urbano

Funding acquisition: Maria Carla Re

Writing – original draft preparation: Maria Carla Re

Writing – review and editing: Maria Carla Re

## References

[1] Kanters S , Vitoria M , Doherty M , et al. Comparative efficacy and safety of first‐line antiretroviral therapy for the treatment of HIV infection: a systematic review and network meta‐analysis. Lancet HIV. 2016;3(11):510‐520. 10.1016/S2352-3018(16)30091-1 27658869

[2] WHO World Health Organization . Guidelines for the use of antiretroviral agents in HIV‐1‐infected adults and adolescents 2017, available at http://aidsinfo.nih.gov/guidelines.

[3] Antinori A , Marcotullio S , Andreoni M , et al. Italian HIV Guidelines Working Group. Italian guidelines for the use of antiretroviral agents and the diagnostic‐clinical management of HIV‐1 infected persons. Update 2015. New Microbiol. 2016;39(2):93‐109. 27196547

[4] Antinori A , Di Biagio A , Marcotullio S , et al. Italian HIV Guidelines Working Group. Italian guidelines for the use of antiretroviral agents and the diagnostic‐clinical management of HIV‐1 infected persons. Update 2016. New Microbiol. 2017;40(2):86‐98. 28513816

[5] Aldous JL , Haubrich RH . Defining treatment failure in resource‐rich settings. Curr Opin HIV AIDS. 2009;4(6):459‐466. 20048711 10.1097/COH.0b013e328331dea5 PMC2946177

[6] Wang S , Rong L . Stochastic population switch may explain the latent reservoir stability and intermittent viral blips in HIV patients on suppressive therapy. J Theor Biol. 2014;7(360):137‐148. 10.1016/j.jtbi.2014.06.042 25016044

[7] Nettles RE , Kieffer TL . Update on HIV‐1 viral load blips. Curr Opin HIV AIDS. 2006;1(2):157‐161. 19372801 10.1097/01.COH.0000203834.24221.13

[8] Do T , Duncan J , Butcher A , Liegler T . Comparative frequencies of HIV low‐level viremia between real‐time viral load assays at clinically relevant thresholds. J Clin Virol. 2011;52(l):83‐89. 10.1016/j.jcv.2011.09.022 21995930

[9] Wojewoda CM , Spahlinger T , Harmon ML , et al. Comparison of Roche Cobas AmpliPrep/Cobas TaqMan HIV‐1 test version 2.0 (CAP/CTM v2.0) with other real‐time PCR assays in HIV‐1 monitoring and follow‐up of low‐level viral loads. J Virol Methods. 2013;187(1):1‐5. 23098667 10.1016/j.jviromet.2012.10.004

[10] Amendola A , Marsella P , Bloisi M , Forbici F , Angeletti C , Capobianchi MR . Ability of two commercially available assays (Abbott RealTime HIV‐1 and Roche Cobas AmpliPrep/Cobas TaqMan HIV‐1 Version 2.0) to quantify low HIV‐1 RNA levels (<1,000 copies/milliliter): comparison with clinical samples and NIBSC working reagent for nucleic acid testing assays. J Clin Microbiol. 2014;52(6):2019‐2026. 24671791 10.1128/JCM.00288-14 PMC4042785

[11] Swenson LC , Cobb B , Geretti AM , et al. International Viral Load Assay Collaboration. International Viral Load Assay Collaboration. Comparative performances of HIV‐1 RNA load assays at low viral load levels: results of an international collaboration. J Clin Microbiol. 2014;52(2):517‐52323. 24478482 10.1128/JCM.02461-13 PMC3911321

[12] Hologic, Inc . Aptima HIV‐1 Quant Dx assay [product information]. 11/2014 Available at: http://stage.hologic.com/products/clinical-diagnostics-and-blood-screening/assays-and-tests/Aptima-hiv-1-quant-dx-assay; Accessed April 8, 2017.

[13] Roche Molecular Diagnostics . COBAS® AmpliPrep/COBAS® TaqMan® HIV‐1 Test, v2.0. [product information]. 7/2010 Available at: http://molecular.roche.com/assays/Pages/COBASAmpliPrepCOBASTaqManHIV-1Testv20.aspx; Accessed April 8, 2017.

[14] Bon I , Ciccozzi M , Zehender G , et al. HIV‐1 subtype C transmission network: the phylogenetic reconstruction strongly supports the epidemiological data. J Clin Virol. 2010;48(3):212‐214. 20400369 10.1016/j.jcv.2010.03.021

[15] Musumeci G , Magnani G , Bon I , et al. HIV‐1 early and late diagnosis in the Emilia Romagna Region (Italy): a three year study. New Microbiol. 2016;39(4):241‐246. 27727402

[16] Garrett NJ , Apea V , Nori A , et al. Comparison of the rate and size of HIV‐1 viral load blips with Roche COBAS TaqMan HIV‐1 versions 1.0 and 2.0 and implications for patient management. J Clin Virol. 2012;53(4):354‐355. 22261125 10.1016/j.jcv.2011.12.024

[17] DHHS Department of Health and Human Services . Panel on antiretroviral guidelines for adults and adolescents. Guidelines for the use of antiretroviral agents in HIV‐1‐infected adults and adolescents. Updated November 13, 2014 Available at: http://aidsinfo.nih.gov/contentfiles/lvguidelines/AdultandAdolescentGL.pdf. (Accessed April 8, 2017).

[18] Antiretroviral Therapy Cohort Collaboration (ART‐CC) , Vandenhende MA , Ingle S , et al. Impact of low‐level viremia on clinical and virological outcomes in treated HIV‐1‐infected patients. AIDS. 2015;29(3):373‐383. 25686685 10.1097/QAD.0000000000000544

[19] Hopkins M , Hau S , Tiernan C , et al. Comparative performance of the new Aptima HIV‐1 Quant Dx assay with three commercial PCR‐based HIV‐1 RNA quantitation assays. J Clin Virol. 2015;69:56‐62. 26209380 10.1016/j.jcv.2015.05.020

[20] Hatzakis A , Papachristou H , Nair SJ , et al. Analytical characteristics and comparative evaluation of Aptima HIV‐1 Quant Dx assay with Ampliprep/COBAS TaqMan HIV‐1 test v2.0. Virol J. 2016;13(1):176. 27769309 10.1186/s12985-016-0627-y PMC5073876

[21] Manak MM , Hack HR , Nair SV , et al. Evaluation of Hologic Aptima HIV‐1 Quant Dx assay on the Panther System on HIV Subtypes. J Clin Microbiol. 2016;54(10):2575‐2581. 27510829 10.1128/JCM.01350-16 PMC5035401

[22] Nair SV , Kim HC , Fortunko J , et al. Aptima HIV‐1 Quant Dx—a fully automated assay for both diagnosis and quantification of HIV‐1. J Clin Virol. 2016;77:46‐54. 26896710 10.1016/j.jcv.2016.02.002

[23] Sahoo MK , Varghese V , White E , et al. Evaluation of the Aptima HIV‐1 Quant Dx assay using plasma and dried blood spots. J Clin Microbiol. 2016;54(10):2597‐2601. 27535684 10.1128/JCM.01569-16 PMC5035416

[24] Sauné K , Raymond S , Boineau J , Pasquier C , Izopet J . Detection and quantification of HIV‐1 RNA with a fully automated transcription‐mediated‐amplification assay. J Clin Virol. 2016;84:70‐73. 27728849 10.1016/j.jcv.2016.09.002

[25] Schalasta G , Börner A , Speicher A , Enders M . Comparative evaluation of the Aptima HIV‐1 Quant Dx assay and COBAS TaqMan HIV‐1 v2.0 assay using the Roche High Pure System for the quantification of HIV‐1 RNA in plasma. Clin Chem Lab Med. 2016;54(3):493‐499. 26351942 10.1515/cclm-2015-0522

[26] Schønning K , Johansen K , Landt B , Benfield T , Westh H . Comparison of the Hologic Aptima HIV‐1 Quant Dx assay to the Roche COBAS Ampliprep/COBAS TaqMan HIV‐1 Test v2.0 for the quantification of HIV‐1 RNA in plasma samples. J Clin Virol. 2017;92:14‐19. 28505569 10.1016/j.jcv.2017.05.006

[27] O'’Shea S , Nair SV , Kim HC , et al. Performance of the Aptima® HIV‐1 Quant Dx Assay on the Panther System. Intl J Med Health Biomed Bioengin Pharma Engin. 2015;9(5):397‐400.

[28] Mor O , Gozlan Y , Wax M , et al. Evaluation of the RealTime HIV‐1, Xpert HIV‐1, and Aptima HIV‐1 Quant Dx assays in Comparison to the NucliSens EasyQ HIV‐1 v2.0 Assay for Quantification of HIV‐1 Viral Load. J Clin Microbiol. 2015;53(11):3458‐3465. 26292298 10.1128/JCM.01806-15 PMC4609691

[29] Amendola A , Pisciotta M , Aleo L , Ferraioli V , Angeletti C , Capobianchi MR . Evaluation of the Aptima(®) HIV‐1 Quant Dx assay for HIV‐1 RNA viral load detection and quantitation in plasma of HIV‐1‐infected individuals: a comparison with Abbott RealTime HIV‐1 assay. J Med Virol. 2016;88(9):1535‐1544. 26864171 10.1002/jmv.24493 PMC6585778

[30] Sam SS , Kurpewski JR , Cu‐Uvin S , Caliendo AM . Evaluation of performance characteristics of the Aptima HIV‐1 Quant Dx assay for detection and quantitation of human immunodeficiency virus type 1 in plasma and cervicovaginal lavage samples. J Clin Microbiol. 2016;54(4):1036‐1041. 26842702 10.1128/JCM.03289-15 PMC4809915

[31] Ratnam S , Jang D , Gilchrist J , et al. Workflow and maintenance characteristics of five automated laboratory instruments for the diagnosis of sexually transmitted infections. J Clin Microbiol. 2014;52(7):2299‐2304. 24740081 10.1128/JCM.03549-13 PMC4097743

